# p42MAPK-mediated phosphorylation of xEIAP/XLX in *Xenopus *cytostatic factor-arrested egg extracts

**DOI:** 10.1186/1471-2091-8-5

**Published:** 2007-04-11

**Authors:** Yuichi Tsuchiya, Shigeru Yamashita

**Affiliations:** 1 Department of Biochemistry, Toho University School of Medicine, 5-21-16 Omori-nishi, Ota-ku, 143-8540 Tokyo, Japan

## Abstract

**Background:**

BIR family proteins are evolutionarily conserved anti-apoptotic molecules. One member of *Xenopus *BIR family proteins, xEIAP/XLX, is a weak apoptosis inhibitor and rapidly degraded in a cell-free apoptotic execution system derived from interphase egg extracts. However, unfertilized eggs are naturally arrested at the metaphase of meiosis II by the concerted activities of Mos-MEK-p42MAPK-p90Rsk kinase cascade (cytostatic factor pathway) and many mitotic kinases. Previous studies suggest that cytostatic factor-arrested egg extracts are more resistant to spontaneous apoptosis than interphase egg extracts in a p42MAPK-dependent manner. We tested whether xEIAP/XLX might be phosphorylated in cytostatic factor-arrested egg extracts, and also examined whether xEIAP/XLX could be functionally regulated by phosphorylation.

**Results:**

We found that p42MAPK was the major kinase phosphorylating xEIAP/XLX in cytostatic factor-arrested egg extracts, and three Ser residues (Ser 235/251/254) were identified as p42MAPK-mediated phosphorylation sites. We characterized the behaviors of various xEIAP/XLX mutants that could not be phosphorylated by p42MAPK. However, neither protein stability nor anti-apoptotic ability of xEIAP/XLX was significantly altered by the substitution of Ser with either Ala or Asp at these three sites.

**Conclusion:**

xEIAP/XLX is physiologically phosphorylated by p42MAPK in *Xenopus *unfertilized eggs. However, this protein may not serve as an essential mediator of p42MAPK-dependent anti-apoptotic activity.

## Background

In various animal species including *Xenopus*, ovulated mature eggs have to survive without the support of surrounding follicle cells until successful fertilization. In contrast with the long life of immature oocytes in ovary, the life of ovulated mature eggs is limited to only a few days. Many reports indicate that aged eggs without fertilization or parthenogenetically activated eggs eventually die by apoptosis [reviewed in [1-3]]. Although the exhaustion of nutrients can contribute to oocyte/egg apoptosis [4], the mechanism of this machinery is still poorly understood.

The translation of Mos protein kinase begins during oocyte maturation and automatically activates Mos-MEK-ERK (p42MAPK in *Xenopus *oocyte)-p90Rsk kinase cascade. This is called CSF (cytostatic factor) pathway because its primary role is to arrest the cell cycle until fertilization [reviewed in [5,6]]. In vertebrates, CSF arrests mature eggs at the metaphase of meiosis II, and many mitotic kinases including Cdc2/cyclin B are also kept active. Recent studies suggest that CSF pathway also regulates apoptosis [7-11], but the exact targets are largely unknown.

Baculovirus IAP repeat (BIR) family proteins are evolutionarily conserved zinc-coordinating proteins, and some members inhibit apoptosis by blocking caspase activities [reviewed in [12,13]]. We recently identified four BIR family proteins in *Xenopus *eggs and examined their apoptosis-inhibiting activities using a cell-free system derived from interphase egg extracts [11]. Whereas xXIAP was a physiological apoptosis inhibitor, xEIAP (identical with XLX reported by Holley *et al*. [14]) only weakly inhibited apoptosis, and neither xSurvivin1/xBIR1 nor xSurvivin2/SIX showed anti-apoptotic activities. However, both CSF and mitotic kinases are inactive in interphase egg extracts, and we wondered whether BIR family proteins might be functionally regulated by phosphorylation in CSF-arrested egg extracts. We found that p42MAPK directly phosphorylated xEIAP/XLX on three Ser residues in the Ser-rich region between BIR2 and RING finger domains in CSF-arrested egg extracts. The effects of phosphorylation on the stability and anti-apoptotic activity of xEIAP/XLX were also examined.

## Results and Discussion

### Phosphorylation-dependent electrophoretic mobility shift of xEIAP/XLX in CSF-arrested egg extracts

**Figure 1:**
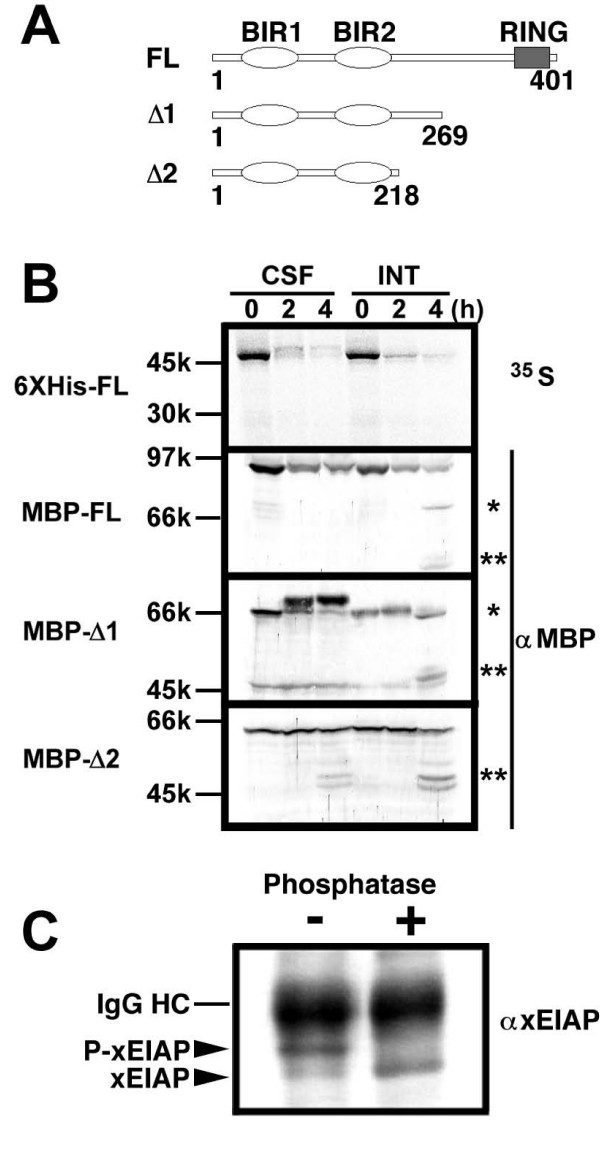
**Phosphorylation-dependent electrophoretic mobility shift of xEIAP/XLX in CSF-arrested egg extracts**. (A) Schematic models of recombinant proteins used in this study. (B) Time-dependent changes of recombinant proteins incubated in CSF-arrested (CSF) and interphase (INT) egg extracts. ^35^S-radiolabeled 6XHis-FL, MBP-FL, MBP-Δ1, and MBP-Δ2 were mixed with either CSF-arrested or interphase egg extracts and incubated for indicated time periods (0, 2, and 4 h). After separation by SDS-PAGE, radiolabeled and MBP-fused recombinants were detected by image analyzer and Western blot, respectively. Molecular weights of standard proteins are indicated at the left. The larger (~70 k) and smaller cleavage products (50–55 k) of MBP-fusions are indicated by single and double asterisks, respectively. (C) Phosphorylation-dependent electrophoretic mobility shift of xEIAP/XLX. Endogenous xEIAP/XLX in CSF-arrested egg extracts were retrieved by antibody-coated beads and incubated with (+) or without (-) calf intestine alkaline phosphatase. After separation by SDS-PAGE, xEIAP/XLX was detected by Western blot. Positions of phosphorylated (P-xEIAP) and unphosphorylated (xEIAP) forms of xEIAP/XLX are indicated by arrowheads. The position of IgG heavy chain (IgG HC) is indicated by the line.

As previously reported, recombinant xEIAP/XLX is rapidly degraded by at least two distinct, consecutively acting proteolytic systems [11,14]. Within 2 h incubation, xEIAP/XLX is significantly degraded in both CSF-arrested and interphase egg extracts in a C-terminal RING finger-dependent manner. Subsequently, spontaneous cytochrome *c*-induced caspase activation begins after 4 h incubation in interphase egg extracts (apoptotic egg extracts), and the remaining xEIAP/XLX is cleaved by the activated caspases at yet unidentified site(s). This caspase activation is delayed or suppressed in CSF-arrested egg extracts by a p42MAPK-dependent pathway [7-11]. We found that the electrophoretic mobilities of recombinant 6XHis-tagged (6XHis-FL) and MBP-tagged (MBP-FL) xEIAP/XLX slightly decreased during incubation in CSF-arrested but not interphase egg extracts (Fig. 1B), whereas those of other BIR family proteins (xSurvivin1/xBIR1, xSurvivin2/SIX, and xXIAP) did not (data not shown). However, the rapid degradation of both 6XHis-FL and MBP-FL in egg extracts hampered the detailed analysis of this shift. Thus, we also used the C-terminally truncated stabilized forms of xEIAP/XLX [11]. MBP-Δ1, consisting of residues 1–269 of xEIAP/XLX, showed a marked upward shift in CSF-arrested but not interphase egg extracts. In contrast, the electrophoretic mobility of MBP-Δ2 consisting of residues 1–218 of xEIAP/XLX did not change in both extracts (Fig. 1B). To confirm that upward shift in CSF-arrested egg extracts was due to phosphorylation, endogenous xEIAP/XLX was immunoprecipitated from CSF-arrested egg extracts and then treated with alkaline phosphatase. As shown in Fig. 1C, the phosphatase treatment increased the electrophoretic mobility of endogenous xEIAP/XLX, indicating that the upward shift was indeed induced by phosphorylation. These results suggest that the region encompassing residues 219–269 of xEIAP/XLX contains CSF-phosphorylation site(s). We also found that, in apoptotic egg extracts, MBP-fused recombinants generated several shorter fragments, most likely produced by caspase-mediated cleavages [11,14]. The larger fragment (~70k, single asterisk) was slightly smaller than MBP-Δ1, whereas the smaller fragments (50–55k, double asterisks) were close to the parental MBP (45k). The cleavage of MBP-Δ2, which was smaller than the larger fragment, produced only the smaller fragments. Our data indicate that xEIAP/XLX is cleaved at several sites, one of which locates between the residues 219–269. It should be noted that our Western blot detected only the MBP-fused N-terminal fragments but not the released C-terminal fragments. We did not observe any cleavage products of radiolabeled 6XHis-FL, suggesting that the generated C-terminal fragments might be further degraded.

### Phosphorylation of xEIAP/XLX by p42MAPK in CSF-arrested egg extracts

**Figure 2:**
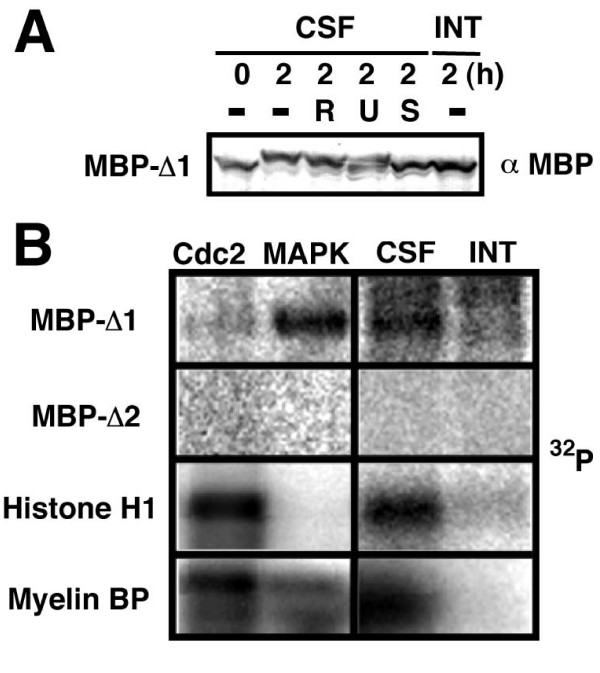
**Phosphorylation of xEIAP/XLX by p42MAPK in CSF-arrested egg extracts**. (A) The effects of kinase inhibitors. MBP-Δ1 was incubated in CSF-arrested (CSF) and interphase (INT) egg extracts, respectively, with or without the indicated kinase inhibitors (R, 100 μM roscovitine; U, 100 μM U0126; S, 10 μM staurosporine) for indicated time periods (0 and 2 h). After separation by SDS-PAGE, MBP-Δ1 was detected by Western blot. (B) Phosphorylation of xEIAP/XLX by p42MAPK and CSF-arrested egg extracts. MBP-Δ1, MBP-Δ2, and control substrates Histone H1 and myelin basic protein (Myelin BP) were incubated with Cdc2/Cyclin B2 (Cdc2), p42MAPK (MAPK), CSF-arrested (CSF) egg extracts, or interphase (INT) egg extracts in the presence of [γ-^32^P] ATP. After separation by SDS-PAGE, incorporated radioactivity was detected by image analyzer.

To identify the kinase(s) phosphorylating xEIAP/XLX, we next tested various protein kinase inhibitors in the assay using MBP-Δ1 as substrate. The presence of 100 μM roscovitine, a specific Cdk inhibitor, could not block the upward shift of MBP-Δ1 (Fig. 2A). In contrast, 100 μM U0126, a specific inhibitor for MEK and thereby an indirect inhibitor for downstream p42MAPK and p90Rsk, partially inhibited the change of electrophoretic mobility. Staurosporine, a general protein kinase inhibitor, completely blocked the shift when supplied to CSF-arrested egg extracts at 10 μM (Fig. 2A). This result indicated that CSF pathway was involved in xEIAP/XLX phosphorylation. It should be noted that the complete inhibition of already activated p42MAPK and 90Rsk requires not only MEK inhibition but also the dephosphorylation of these kinases. The partial inhibition by U0126 suggests that xEIAP/XLX may be phosphorylated by downstream p42MAPK and/or p90Rsk rather than MEK itself. Indeed, recombinant p42MAPK phosphorylated MBP-Δ1, but not MBP-Δ2, *in vitro *(Fig. 2B, MAPK). In contrast, neither substrate was phosphorylated by immunoprecipitated Cdc2/Cyclin B2 complex (Fig. 2B, Cdc2). In control reactions, p42MAPK selectively phosphorylated myelin basic protein but not histone H1, whereas Cdc2/Cyclin B2 strongly phosphorylated both substrates. Similarly, MBP-Δ1, histone H1, and myelin basic protein, but not MBP-Δ2, were phosphorylated in CSF-arrested (Fig. 2B, CSF) but not interphase egg extracts (Fig, 2B, INT). Altogether, we conclude that xEIAP/XLX is directly phosphorylated by p42MAPK in CSF-arrested egg extracts.

### p42MAPK phosphorylates Ser235/251/254 of xEIAP/XLX

**Figure 3:**
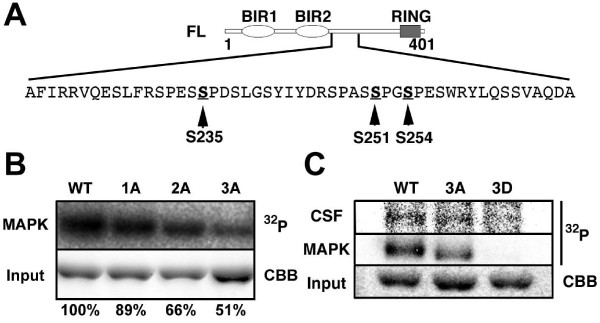
**p42MAPK phosphorylates Ser235/251/254 of xEIAP/XLX**. (A) Amino acid sequence of xEIAP/XLX residues 219–269. Indicated three Ser residues are changed to either Ala or Asp. (B) Phosphorylation of WT, Ser235Ala single mutant (1A), Ser251/254Ala double mutant (2A), and Ser235/251/254Ala triple mutant (3A). (C) Phosphorylation of WT, 3A, and Ser235/251/254Asp triple mutant (3D). In (B) and (C), MBP-Δ1 variants were incubated with CSF-arrested egg extracts (CSF) or p42MAPK (MAPK) in the presence of [γ-^32^P] ATP. After separation by SDS-PAGE, incorporated radioactivity was detected by image analyzer. Same amounts of substrates were also stained with Coomassie Brilliant Blue (Input). In (B), the phosphorylation intensities were calculated using NIH ImageJ software and indicated below.

The optimal sequence for MAPK-mediated phosphorylation is Pro-Xaa-(Xaa)-Ser/Thr-Pro and the minimal sequence requirement is Ser/Thr-Pro. There are five Ser-Pro-Xaa-Ser repeats within the residues 219–269 of xEIAP/XLX, three of which (Ser235/251/254) fulfil optimal MAPK consensus (Fig. 3A). Although another optimal MAPK consensus site resides inside the first BIR domain of xEIAP/XLX (Ser58), this residue may not be recognized because MBP-Δ2 was not phosphorylated by p42MAPK (Fig. 2B). As C-terminal region absent in MBP-Δ1 (residues 270–401) contains no MAPK consensus site, MBP-Δ1 is a suitable substrate to study the phosphorylation of xEIAP/XLX by p42MAPK. We first tried to evaluate the phosphorylation of individual Ser residues by Ala substitution, with closely located Ser251 and Ser254 mutated together. We analyzed the p42MAPK-mediated phosphorylation of WT, Ser235Ala single mutant (1A), Ser251/254Ala double mutant (2A), and Ser235/251/254Ala triple mutant (3A). As shown in Fig. 3B, 1A and 2A mutations led to 11% and 34% reductions of phosphorylation, respectively. The decrease of phosphorylation in 3A mutant (49%) was close to the sum of decreases in 1A and 2A mutants (45%), suggesting that these residues were independently phosphorylated. However, 3A mutant was still significantly phosphorylated, probably at Ser231/247, both in CSF-arrested extracts and by recombinant p42MAPK (Fig. 3C). Interestingly, when the same three Ser residues were changed to Asp (3D), this mutant was not phosphorylated at all (Fig. 3C). The most straightforward interpretation of these data may be that Ser235/251/254 are the primary residues targeted by p42MAPK. The remaining residues were phosphorylated in 3A mutant but not in 3D mutant, presumably because the net charges also affected the phosphorylation status of xEIAP/XLX. The introduction of negative charges by p42MAPK-mediated phosphorylation may be limited to at most three sites under physiological condition, and remaining two residues can be phosphorylated only when Ser235/251/254 are substituted to neutral residues.

### Phosphorylation of Ser235/251/254 affects neither protein stability nor apoptosis-inhibiting activity of xEIAP/XLX

**Figure 4:**
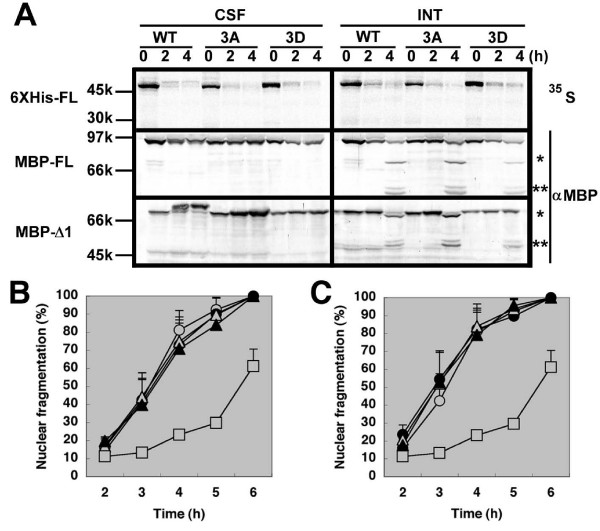
**Phosphorylation of Ser235/251/254 affects neither protein stability nor apoptosis-inhibiting activity of xEIAP/XLX**. (A) Stability and electrophoretic mobility shift of xEIAP/XLX. The WT/3A/3D forms of ^35^S-radiolabeled 6XHis-FL, MBP-FL, and MBP-Δ1 were incubated in CSF-arrested (CSF) or interphase (INT) egg extracts for indicated time periods (0, 2, and 4 h). After separation by SDS-PAGE, radiolabeled and MBP-fused recombinants were detected by image analyzer and Western blot, respectively. Molecular weights of standard proteins are indicated at the left. The larger (~70 k) and smaller cleavage products (50–55 k) of MBP-fusions are indicated by single and double asterisks, respectively. (B) Apoptosis inhibition by MBP-FL. (C) Apoptosis inhibition by MBP-Δ1. In (B) and (C), nuclear fragmentation assays were carried out as previously described [11]. Open circles, MBP (negative control); closed circles, WT; open triangles, 3A; closed triangles, 3D; open squares, MBP-xXIAP (positive control). Data are represented as mean + S.E.M. (N = 5).

We next tested whether the substitutions of p42MAPK phosphorylation sites could affect the electrophoretic mobility, protein stability, and anti-apoptotic functions of xEIAP/XLX. For 6XHis-FL, MBP-FL, and MBP-Δ1, the upward band shift of WT observed in CSF-arrested egg extracts was reduced in 3A mutants and blocked in 3D mutants (Fig. 4A). Moreover, the larger cleavage product (single asterisk) in apoptotic egg extracts was slightly decreased in MBP-FL 3D mutant and absent in MBP-Δ1 3D mutant. If we assume that this fragment is produced by caspase-mediated cleavage after Asp, the cleavage site may be Asp237 and the introduction of negative charge at Ser235 may inhibit caspase recognition especially in MBP-Δ1. However, the smaller fragments of MBP-FL and MBP-Δ1 (double asterisks) were equally observed in all the three recombinant forms. Thus, although MBP-Δ1 was stabilized by 3D mutation against caspase-mediated cleavage in apoptotic egg extracts, the overall stabilities of 6XHis-FL and MBP-FL were not significantly altered by 3A or 3D mutation in both egg extracts, indicating that the alterations of p42MAPK recognition sites did not modify the stability of xEIAP/XLX in egg extracts. Next, we asked whether the apoptosis-inhibiting activity of xEIAP/XLX is affected by the same substitutions. As previously described, the time-dependent fragmentation of sperm nuclei is the hallmark of apoptosis execution in interphase egg extracts [11,16-18]. The addition of MBP-xXIAP strongly inhibited nuclear fragmentation (positive control) compared to non-fused MBP (negative control). All xEIAP/XLX recombinants did not inhibit apoptosis significantly in our experiments, either in the presence (MBP-FL; Fig. 4B) or absence (MBP-Δ1; Fig. 4C) of RING finger-containing C-terminus. Similar results were obtained in CSF-arrested egg extracts, as judged from the caspase-dependent cleavage of endogenous p150^Glued ^at 6–8 h (nuclear morphology could not be analyzed because of mitotic chromatin condensation, data not shown). Moreover, we previously reported that the immunodepletion of xEIAP/XLX from either CSF-arrested or interphase egg extracts showed no effect on apoptotic execution [11]. Altogether, we conclude that neither protein stability nor apoptosis-inhibiting activity of xEIAP/XLX is affected by p42MAPK-mediated phosphorylation.

## Conclusion

Our data indicate that, although xEIAP/XLX is a physiologically phosphorylatable substrate for p42MAPK, it may not be a direct mediator of p42MAPK-dependent anti-apoptotic activity in CSF-arrested egg extracts. One possible role of xEIAP/XLX might be to titrate or ubiquitylate pro-apoptotic molecules, thereby indirectly supporting the anti-apoptotic role of xXIAP [19-23]. Otherwise, xEIAP/XLX could regulate the abundance of xXIAP [24-26] or ubiquitylate apoptotic signal transducers [22,27-30]. Further studies to address these issues are currently in progress. During the preparation of this manuscript, Greenwood and Gautier also reported that xEIAP/XLX is phosphorylated mainly by MAPK during meiosis [31].

## Methods

### Preparation of recombinant proteins

Vector construction, bacterial expression and affinity purification of maltose binding protein (MBP)-fused recombinant proteins were previously described [11]. *In vitro *translation of ^35^S-radiolabeled 6XHis-tagged recombinant proteins in rabbit reticulocyte lysates using TnT T7 Quick (Promega, Tokyo, Japan) and Pro-Mix (GE Healthcare, Tokyo, Japan) was carried out according to manufacture's instructions. Site-directed mutagenesis was performed using QuikChange (Stratagene, CA, USA) and confirmed by DNA sequencing.

### Preparation of *Xenopus *egg extracts

Preparations of CSF-arrested, interphase, and apoptotic egg extracts were previously described [11,15-18]. Where indicated, roscovitine (Calbiochem-Merck, Tokyo, Japan), U0126 (Sigma-Aldrich, Tokyo, Japan), and staurosporine (Sigma-Aldrich) were supplied to egg extracts.

### Protein stability assay

MBP-fused recombinants were added to egg extracts at 1 μg/ml, whereas rabbit reticulocyte lysates containing radiolabeled recombinants were mixed with egg extracts at 1:9. After incubation, samples were resolved by SDS-PAGE, and remaining MBP-fused and radiolabeled recombinants were detected by Western blot using anti-MBP antiserum (New England Biolabs, MA, USA) and by BAS-5000 image analyzer (Fuji Film, Tokyo, Japan), respectively.

### Protein phosphorylation assay

Recombinant activated p42MAPK was purchased from New England Biolabs. Active Cdc2/Cyclin B2 complex was immunoprecipitated from CSF-arrested egg extracts by affinity-purified anti-*Xenopus *Cyclin B2 antibody immobilized on Affi-Prep Protein A beads (Bio-Rad, Tokyo, Japan). Histone H1 and myelin basic protein were purchased from Roche (Tokyo, Japan) and Sigma-Aldrich, respectively. Kinase assay using purified p42MAPK and Cdc2/Cyclin B2 was performed in 10 μl of Assay Buffer (10 mM HEPES-KOH, pH 7.7, 15 mM MgCl_2_, 1 mM DTT) containing respective kinase, 1 μg of substrate protein, 100 μM cold ATP, and 37 kBq of [γ-^32^P] ATP (GE Healthcare). For phosphorylation assay by egg extracts, either CSF-arrested or interphase egg extracts were first diluted 5-fold with MEB-TX (20 mM HEPES-KOH, pH 7.7, 15 mM MgCl_2_, 80 mM sodium glycerol 2-phosphate, 20 mM EGTA, 1 mM DTT, 0.2 mM phenylmethanesulfonyl fluoride, 0.1% Triton X-100). Diluted extracts were then supplied with 1 μg of substrate protein and 37 kBq of [γ-^32^P] ATP in 10 μl. After the reaction at 30°C for 30 min, samples were resolved by SDS-PAGE and analyzed with image analyzer.

### Dephosphorylation of endogenous xEIAP/XLX

Affinity-purified anti-xEIAP/XLX antibody was first immobilized on Affi-Prep Protein A beads and then mixed with CSF-arrested egg extracts to retrieve endogenous xEIAP/XLX. After extensive washing, the xEIAP/XLX-loaded beads in Assay Buffer were incubated with calf intestine alkaline phosphatase (Roche) overnight at 30°C, followed by resolution by SDS-PAGE and Western blot with the same antibody.

### Apoptosis inhibition assay

Recombinant proteins were added to interphase egg extracts at 10 μg/ml, and apoptotic nuclear fragmentation was observed as previously described [11].

## Competing interests

The author(s) declare that they have no competing interests.

## Authors' contributions

YT designed the study, carried out all experiments, analyzed the data, and drafted the manuscript. SY discussed the data and drafted the manuscript. Both authors read and approved the final manuscript.
